# Negotiating the transition from adolescence to motherhood: Coping with prenatal and parenting stress in teenage mothers in Mulago hospital, Uganda

**DOI:** 10.1186/1471-2458-8-83

**Published:** 2008-03-04

**Authors:** Dan K Kaye

**Affiliations:** 1 Makerere University Medical School, Dept. of Obstetrics and Gynecology, P.O. Box 7072, Kampala, Uganda

## Abstract

**Background:**

Adolescence is a transitional stage from childhood to adulthood that is characterized by physical, physiological, psychosocial and behavioral changes that are influenced to a large extent by the age, culture and socialization of the individual. To explore what adolescent mothers perceive as their struggles during the period of transition from childhood to parenthood (through motherhood) and to describe strategies employed in coping with stress of pregnancy, motherhood and parenthood.

**Methods:**

Longitudinal qualitative study involving twenty two in-depth interviews and six focus group discussions among pregnant adolescents who were followed from pregnant to delivery, from January 2004 to August 2005. Participant were selected by theoretical sampling and data was analyzed using grounded theory.

**Results:**

Overall, young adolescents reported more anxiety, loss of self esteem (when they conceived), difficulty in accessing financial, moral and material support from parents or partners and stigmatization by health workers when they sought care from health facilities. Three strategies by which adolescent mothers cope with parenting and pregnancy stress that were described as utilizing opportunities (thriving), accommodating the challenges (bargaining and surviving), or failure (despairing), and varied in the extent to which they enabled adolescents to cope with the stress.

**Conclusion:**

Adolescents on the transition to motherhood have variable needs and aspirations and utilize different strategies to cope with the stress of pregnancy and parenthood.

## Background

Adolescence is a transitional stage from childhood to adulthood that is characterized by physical, physiological, psychosocial and behavioral changes that are influenced to a large extent by the age, culture and socialization of the individual. Programmes for adolescents often fail to recognize the heterogeneity and widely differing needs of the group [1,2], which are influenced by gender roles, age, residence and prior socialization [3,4]. Adolescent relationships are characterized by gender power imbalances, irrational decision-making, poor communication, inadequate preparation for responsibilities of marriage and childbearing, fear of rejection and outside influence from family, peers or partners [3,4].

Research employing qualitative methods (to explore coping with pregnancy and motherhood) may provide the necessary information for informing policy and practice for care of adolescents. Firstly, it provides information on experiences, significance, consequences and implications of adolescent behavior from the perspective of the adolescents themselves [1,2]. Secondly, it may identify (perceived or actual) determinants of negative behavior that are potentially modifiable through interventions [5-7]. Exploring adolescents' experiences provides insights into adolescents' decision-making. Thirdly, analysis of theoretical models for coping with stress may suggest acceptability and likely cost-effectiveness of interventions for pregnancy adolescents or adolescent mothers [8,9]. Behaviors that adolescents adopt in response to the stress of motherhood and childrearing may affect their health, consequently influencing their motherhood experiences as well as exposing their children to avoidable risks such as child neglect or child abuse. The general objective was to explore what adolescents perceived as their struggles during the period of transition from childhood to parenthood and specifically, describe strategies employed in coping with stress of pregnancy, motherhood and parenthood.

## Methods

### Setting and participants

This study was carried out in Mulago hospital, the national referral hospital in Kampala, Uganda from January 2004 to August 2005, in a cohort of pregnant women recruited from the antenatal clinic and followed up to delivery and postnatal clinic. Of the women who attend the hospital's antenatal clinic, about 40% are adolescents, several of whom already have children. The data is derived from twenty two in-depth interviews and six focus group discussions (involving 52 adolescent women). The participants were interviewed during pregnancy and within the first six weeks after delivery. The focus groups were conducted after delivery, and were arranged according to two age groups: those aged 16 years and less and those more than 16 years. Theoretical sampling was used for participant selection: after each interview, data was evaluated to decide next interviewee or which issues to explore further in the focus groups, until data saturation. Interviews carried out in English or Luganda (a local dialect) lasted 45 to 60 minutes and were tape recorded. Ethical clearance to carry out the study, including permission to conduct research on adolescents who are minors, was obtained from Makerere University Higher Degrees Committee, Karolinska Institutet Ethics Committee, Mulago Hospital and Uganda National Council of Science and Technology. Participants gave written informed consent before participation in the study, and were offered psychological counseling for domestic violence and given health education on how to improve personal safety. Those who needed further counseling were referred to professional counselors.

### Theoretical framework

The study employed the stress and coping model [8] to explore how adolescents negotiate the transition from childhood to parenthood in the context of domestic violence. This model suggests that diminished psychological and social coping resources of adolescent mothers influence negative behaviors and result in ineffective parenting behaviors, thus increasing the likelihood of high risk outcomes for both parents and children. The theoretical framework is adapted from studies of South African adolescents [10], based on three premises: Firstly, decision-making is contextual. Secondly, understanding what dictates adolescents' decision-making is key to understanding how, why and which adolescents have risky behavior. Thirdly, understanding social phenomena requires exploration of perceptions, aspirations, actions, attached meanings and interpretations from the perspective of adolescents themselves.

### Data analysis

Grounded theory was employed for data analysis. Using Easy Text software for data retrieval, analysis involved an inductive process of developing codes (open coding) according to key concepts from transcripts and (field) notes. Related emerging codes were identified by selective coding and grouped into categories by constant text comparison. Some codes were descriptive of the coping strategy employed, others were interpretive (the desired consequence of the strategy) or explanatory (reason why the chosen strategy was employed and perception of whether it was successful).

## Results

The participants' age ranged from 14 to 19 years. Only three of those interviewed were in formal employment. Twenty three participants had no formal education, and only five had gone up to secondary level of education. All reported that the relationships that led eventually to the conception were consensual. Only four were in marital relationships which had been formalized or legally recognized, though another six reported that they were cohabiting (living with their partners/boyfriends with the knowledge of their parents or other close relatives). They reported anxiety, loss of self esteem (when they conceived), difficulty in accessing financial, moral and material support from parents or partners and stigmatization by health workers when they sought care from health facilities. There was no major distinction in coping mechanisms according to age group of the adolescents. Analysis of the data revealed three major themes: Utilizing opportunities for change (thriving), accommodating the challenges while tolerating the abandonment of support (bargaining and surviving), or failure to handle the stress in their lives to such an extent that they were overwhelmed by the struggle(despairing).

### Utilizing opportunities for change

Overall, motherhood was a positive experience for the younger and older adolescents, and was looked upon with pride and joy for most adolescents interviewed and those in the focus group discussions. To many adolescents, it was apparent that they had partly achieved what was their heart's desire. This is exemplified by one 18-year-old participant who was having her second pregnancy:

"I was (at the beginning) dismissed from school. My brother (with whom I was staying with) sent me away when I became pregnant, saying that this will embarrass his family as he was a preacher... It is important that you get your children when you are young and strong.... I (later) stayed with my grandmother in the village. Life (then) was not easy. (When) I moved in with my boyfriend, he put me in his shop (where I worked) until I delivered (the first child). (Later) we got married, .... and he gave me money to enroll for a computer course and secretarial work at Y.W.C.A. I (now) have a good job stay with my young sister. He (my husband) pays my school fees."

Whereas some adolescents were eager to settle down into parenting and childrearing roles, some adolescent mothers had high hopes of returning to school once they have given birth. Some had no immediate plans to settle down in marriage, and were ready to go back to school as long as there was someone ready to look after their child, especially if it was their parents or one of the relatives. With such optimism, they were willing to nurse their children and cope with the stress of pregnancy, childbirth and parenthood. This optimism is exemplified by one 16-year old mother:

*"It (getting pregnant) was a mistake*... *I do not plan to marry. I (think) I will go back to school... I will have to change to a new school. I don't think they will accept me back (in my former school). I even got some notes (from some friends) and will go for coaching (if this is necessary). ...(Probed on who will look after the child): My mother is ready to take care of my child. My father will get for me fees. I (promised them) will not be involved in this (relationship) again. In fact they have already secured me a place in a day school, .... and I don't care whether he gets someone else."*

The findings demonstrate that the optimistic coping style (emotion-focused) was frequently used as an effective coping style for the stress of pregnancy and motherhood in these women. From the focus group discussions, such optimistic approaches suggested lack of understanding of the challenges that pregnancy, childbirth and motherhood will place upon them. Participants acknowledged that some adolescents settle down to serious studies after pregnancy, arguing that they have "learnt their lessons", "have no need to adventure", "are more mature and understanding of the world". Such successful participants can be described as thriving despite the adolescent pregnancy and parenthood. The acknowledgement/acceptance of the adolescent pregnancy, moral support to the adolescent mother and material/financial support to look after the child, were important factors that minimized the adolescents' stress and enabled them to cope adequately with pregnancy and subsequent motherhood.

For some adolescents, it was apparent that the pregnancy was planned, and even where it was unplanned, financial, relationship and security were assured, guaranteed or expected. Such adolescents described their relationships as "stable" or "strong", and expressed positive attributes of an adolescent pregnancy and motherhood, as exemplified by one 19-year-old participant from one focus group who was not staying with the partner:

"*I am happy where I am. I do not regret getting pregnant. Actually he (the man) advised on family planning but I refused (for no particular reasons). I am now in a stable relationship (though he has another family). I am happy with my two children even though most of the time he(the child's father)is not around. But (he is a soldier) so I understand his situation even when he does not send (financial) support. ...I wouldn't advise anyone to get children when they are too old, and I don't regret."*

### Accommodating and enduring

Traditional views of adolescent mothers perpetuate negative stereotypes and fail to acknowledge many who seem to cope adequately and provide care for their children despite the challenges. The acknowledgement/acceptance of the adolescent pregnancy, moral support to the adolescent mother and material/financial support to look after the child were perceived to be dependent on paternal recognition. As long as the fathers of the children acknowledged and accepted the paternity, the parents/relatives were willing to look after the children of adolescent mothers so that they could go back to school.

Paternity acceptance was also a preliquisite for the adolescent mothers to move into a more permanent relationship with the father of the child. Unfortunately, some boyfriends/partners were reportedly unwilling to admit paternity (for three of the babies of adolescent mothers interviewed). From focus groups, participants acknowledged that this problem was common and pervasive. Some of the reasons why it was common were that it (admitting paternity) jeopardizes boys' educational and employment opportunities. Other participants thought it was because partners (and their parents) were irresponsible. This was a major source of stress to the adolescent mother, as described by a 17-year-old mother, who reported rejection of the pregnancy and the baby by her boyfriend's family:

"It is hard to accept (that the father has denied responsibility) because they (his or my parents) can't look after you and the child when pregnant. (Probed on why this is so) Sometimes they are forced to deny (responsibility) by circumstances. (They)...fear arrest and imprisonment and may be discontinued (from school) so that (both) your education may end. But yours has also ended and you have no support so what are you expected to do? Sometimes you have nothing. Even the breast milk may not be adequate and you don't have what to feed the baby."

From the interviews and focus group discussions, many teenage mothers reported renewed vigor, strength and hope in with the birth of the children, despite despair during pregnancy. Motherhood and parenting roles were described as "satisfying" and "fulfilling", as it gave adolescents new identity and status. In others, the adolescents satisfaction was reduced by coexisting burdens such as waking up to nurse the baby, having no assistance with looking after the baby, or rebuke by health workers, relatives and even strangers who disapproved of adolescent pregnancy in particular or adolescent sexual relationships in general. Some felt that most people they interacted with considered them unsuitable to be parents by virtue of their age, and experienced. stigmatization. In focus groups discussions, adolescents felt they got inadequate social, moral, material and financial support from their relatives or their partners, and health workers were singled out as a group that has negative attitudes to pregnant adolescents and mothers. To such mothers, adolescent pregnancy and motherhood was big but bearable burden, and were willing to make sacrifices to succeed in their new roles.

Some adolescents described stories of unhappiness in adolescence which extended through pregnancy into motherhood, disrupted lives, turmoil during adolescence and a need to find love and connection in their lives (which they had partly achieved from the adolescent relationships and subsequent pregnancy and motherhood). Unfortunately for some, this happiness was short-lived, as described by a 16 year old mother:

"At the beginning I did not care about what people said, though I was worried about my safety and that of my baby. My boyfriend was not working, so (though) we had little (but) we shared (what we had). But when (my boyfriend) changed, this affected me as we could no longer understand each other. ...we were always quarreling and fighting. I (at times) regretted my (stubborn) behavior (of getting involved in groups and getting boyfriends) which had landed me in this (trouble). I did not know that he could change because he showed me a lot of love and (actually) things were alright (at the beginning) before they (misunderstandings) started. Now (though) I don't have any where to go (for help, the baby is a girl who does not need much. I can buy clothes for her. ... I have adequate breast milk... (I hope) we will survive."

Such adolescents indicated a surprising level of maturity and commitment to pregnancy, motherhood and adolescent parenthood. Expectedly, these mothers described motherhood and parenthood as a positive force that helped change their lives to a more productive and hopeful future, despite their stress and struggles. To some adolescents, conception and motherhood provided acceptance and recognition by the partners' family, which improved their self worth and esteem. This is exemplified by an 18-year-old mother who was interviewed during pregnancy and six weeks after delivery:

Interview before delivery: *"It is hard to leave because you can not stay alone and look after yourself when pregnant. ....My parents and relatives rejected me and they (his people) don't like me. I don't think they like me. I don't think they will change....Sometimes you are forced to go back (to the boyfriend to ask for help) by circumstances, but would not if you could survive on your own."*

Interview after delivery: *Such (being neglected and lacking financial support) has not happened since delivery..... My concern now is to settle down with him and his family. They (he and his family) have been giving me (financial and emotional) "support", which was not the case (before becoming pregnant or delivery). I think they like (me and) the baby and we will look after the baby (together with them)."*

## Failing and despairing

While most adolescents seemed to cope with the stress of the transition from adolescence to parenthood, some seemed unable to cope. Some participants felt nearly overwhelmed by the heavy burden of stigma due to the adolescent pregnancy and were just struggling to survive. Such adolescents expressed regrets that pregnancy reduced their opportunities in life, and regretted the decision to get into relationships, conceive or go into motherhood at an early age. Three participants strongly regretted having kept the pregnancy in the initial interview during pregnancy (and two of these had ambivalent feelings about the newborn in the subsequent interval at birth), which cast doubt about the future safety of the newborns. They further reported difficulties in breastfeeding and bonding with the children and their babies were apparently underweight or malnourished. To such women, the transition from adolescence to motherhood was a bumpy road with few episodes of happiness or satisfaction, to an extent that they appeared desperate, overwhelmed by the stress of pregnancy and subsequent motherhood. This is exemplified by one of them, a mother of three, who despite moving into a marital home with the father of her child at 15 years, found married life, parenthood and child rearing difficult and on many occasions 'unbearable":

"It is hard to survive because you can't look after yourself and the baby. (I) can't even get a job as you are not educated. You have nobody to help you and nowhere to turn. ... could survive on odd jobs but what you get is not enough. I have nowhere to leave the child and feeding it is a problem. (I understand why) sometimes some women abandon their babies, (as you are) forced to by circumstances."

For some adolescents in the focus groups, their view of motherhood and childrearing were negative and to some extent could be described as "hostile". Hostile childrearing attitudes contributed to increased stress in the adolescent mothers. From discussion on what could be responsible for such negative perceptions and what were likely consequences of such behavior, a pattern emerged, suggesting that those with more hostile attitudes engaged in behavior that contributed directly to making their lives more stressful. Such behavior also made it difficult for them obtaining the moral and financial support they needed, subsequently making it difficult for them to look after their children successfully. Some of the behaviors described included having multiple sex partners, alcohol abuse and drug abuse (often starting before they conceived or extending into the postpartum period).

Risk-taking was also influenced by or associated with lack of security and safety in daily lives, emotion-focused coping and peer pressure. Such a combative coping style (problem-focused) was also used and found to be effective by some adolescents, as exemplified by one participant, a 17-year-old single mother, who seemed overwhelmed by the stress of adolescence, pregnancy, motherhood and parenthood:

"I think it was wrong to marry (at an early age) and I am now regretting. I only stayed (with him) because I am not sure what to do or where to go. I (now) have someone else who is the not father of the child (pregnancy) though he is not aware. Some (of my friends) have advised me to leave (him). (The most important thing is that) it is my child I cannot leave my child to suffer."

## Discussion

In many developing countries especially in sub-Saharan Africa, adolescent pregnancy is readily identified as one of the pressing social and reproductive health issues. However, this perception is rarely translated into programmes that effectively reach adolescents' needs. This may be due to lack of awareness of their specific needs. This study that explored experiences and coping strategies of adolescents on the transition from adolescence to parenthood indicates that many adolescents take on adult roles and responsibilities when they are ill-prepared. This affects adolescents to different extents. The study shows that teenage pregnancy and parenthood may be socially accepted as a source of identity and status and may be prestigious to some adolescents. While they may be undesirable and strongly resented but tolerated in some adolescents, they may markedly reduce the quality of life of those affected in others.

The findings are in agreement with other studies on adolescents in both developed [11-13] and developing countries such as Uganda [14]. All these studies indicate that adolescent behavior is often dependent on the social needs and physical as well as psychological level of development, which likewise are largely influenced by prior and current experiences and aspirations. Pregnancy among adolescents in sub- Saharan African countries such as Uganda is often viewed as a social problem, especially when it occurs out of marital relationships [15]. This is because such pregnancy interferes with expectations regarding educational prospects, self-realization, marital prospects or economic prosperity [15].

The findings are in agreement with Varga (2003) that gender roles are important contextual determinants of the decision-making process for adolescents. Secondly, psychological factors may influence risk taking behaviour in young adults, a finding that has been observed even in developed country contexts such as Australia and Sweden [11-13]. They further indicate that the coping style for stress of the transition from adolescence to motherhood is a combination of coping with pregnancy [16] and coping with adolescence [17]. This finding has been noted by researchers on cohorts of adolescents in Sweden and Australia [11-13]. The coping mechanisms employed combine emotion focused and problem-focused approaches, with predominance of the former. Adolescents are a heterogeneous group with regard to goals, aspirations and social needs. The needs of adolescents are variable at different stages of the transition and therefore care needs to be individualized, in order to meet the goals of the adolescents, in agreement with prior studies in American adolescents [12,18]. In the conservation of resources theory, Hobfoll et al [19] stress occurs in situations where resources (such as time, emotions, material resources) are lost, threatened or invested without gain. The study findings indicate that unmet expectations associated with financial and material deprivation were sources of stress to the adolescents.

The implication study's findings is that healthcare providers need to recognize that pregnant adolescents and adolescent mothers have varying needs. They should carefully assess each individual's strengths, weaknesses, hopes, and goals prior to developing a plan of care. They also need to design relevant and appropriate strategies to reduce adolescents' stress or offer relevant health education to enable them cope with stress of pregnancy and motherhood. Secondly, adolescent programs need to be flexible in order to be responsive to the changing needs of the adolescents as they negotiate the transition from adolescence to motherhood and parenthood.

## Conclusion

Adolescents on the transition to motherhood have variable needs and aspirations, and therefore use different strategies to cope with the stress of pregnancy and parenthood. The stress and coping model can be used to explain and interpret the behavior exhibited by adolescents as they negotiate this transitional period.

## Competing interests

The author(s) declare that they have no competing interests.

## Authors' contributions

DKK was involved in the conceptualization and design of the study, design of the interview instruments, data collection, data analysis, manuscript writing and revision of the manuscript at all its stages.

## Pre-publication history

The pre-publication history for this paper can be accessed here:


